# Reversed cross finger subcutaneous flap: A rapid way to cover finger defects

**DOI:** 10.4103/0970-0358.41112

**Published:** 2008

**Authors:** Nawfal Fejjal, Redouane Belmir, Samir El Mazouz, Noureddine Gharib, Abdellah Abbassi, Amin Belmahi

**Affiliations:** Plastic Surgery Unit, IBN SINA Hospital, Rabat, Morocco

**Keywords:** Homodigital flaps, finger flaps, adipofascial flaps, defect

## Abstract

Adequate coverage of dorsal finger wounds is often a challenge. The reversed cross finger subcutaneous flap to cover defects on the dorsum of phalanx constitutes an excellent option for coverage of wounds over the middle and distal phalanges of the index, middle, ring, and small fingers. It's an easy flap and represents our first choice to cover those defects.

## INTRODUCTION

**Figure 1:**
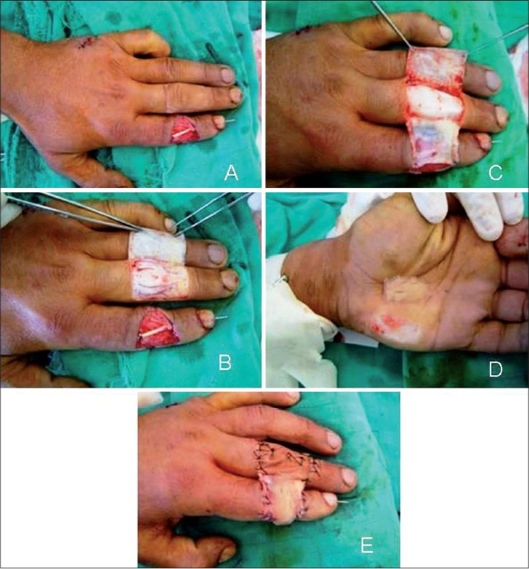
(A) Design of the flap: we can see the tendon graft. (B) De epithelialisation. (C) The reversed cross finger subcutaneous flap is raised up and sutured to the recipient site. (D) The full thickness skin graft from the hypothenar region. (E) Aspect at the end of the operation

**Figure 2:**
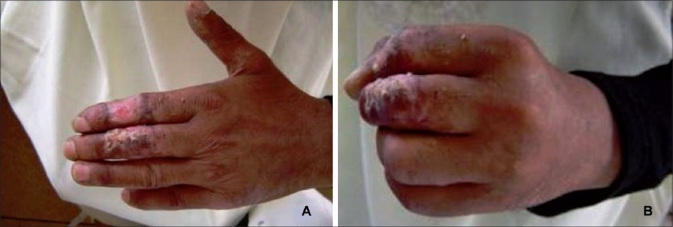
Aspect at one month after the operation. Good functional result at flexion and extension

We report here a case of a butcher who injured his left index while cutting meat. This trauma was responsible for a defect involving the extensor system and the skin at the level of the middle phalanx. The patient was operated under loco-regional anaesthesia. The proximal and distal interphalangeal joints were fixed in extension using kirschner wire. The extensor system was repaired using a tendon graft from the extensor proprius tendon of the little finger. The skin defect was covered using a reversed cross finger subcutaneous flap harvested from the dorsum of the third finger. The deep side of the flap was grafted after fixation in the recipient site using a full thickness skin graft from the hypothenar region. The flap division was done in the third week and the patient recovered good function both in flexion and extension [Figures 1-2].

Adequate coverage of dorsal finger wounds is often a challenge for surgeons. Adipofascial flaps constitute an excellent option for many reasons: thinness, good pliability, minimal donor site deformity and the simplicity and rapidity of the procedure.

The harvesting of these flaps is possible because dorsal cutaneous branches from the proper palmar digital artery supply the dorsum of the finger. There are several studies showing that these vessels are constant over the proximal and middle phalanges.[1]

Many authors describe the reversed cross finger subcutaneous flap or random-pattern deepithelialised flap[2–4] to cover defects on the dorsum of phalanx.[5–9] The design of the flap is made exactly following the limits of functional phalanx unit. For easy dissection, we prefer to infiltrate the flap using physiological serum. The procedure begins by making proximal, distal and midlateral incisions on the side of the defect [Figures 1A-B].

Proximal, distal and lateral incisions in the sub cutis are carried out and the flap is raised including all tissue between the dermis and the paratenon [Figure 1C]. The flap is then turned laterally on its attached base to reach the opposite side of the defect. After the flap is fixed to the defect, the skin over the donor site is repositioned over the paratenon and a split-thickness skin graft is applied to the raw surface of the reversed flap [Figures 1D-E].

The flap division can be made from the 7^th^ to the 12^th^ day because of the excellent revascularisation of the flap.[9] In our experience, we prefer to do it from the 15^th^ to the 21^st^ day for greater vascular security.

The reversed cross finger subcutaneous flap has appeared as an excellent alternative for achieving early coverage of cutaneous wounds at the dorsal aspect of proximal and middle phalanges of the long fingers. It's an easy flap and represents our first choice to cover those defects.
